# Biological Connection of Psychological Stress and Polytrauma under Intensive Care: The Role of Oxytocin and Hydrogen Sulfide

**DOI:** 10.3390/ijms22179192

**Published:** 2021-08-25

**Authors:** Tamara Merz, Oscar McCook, Nicole Denoix, Peter Radermacher, Christiane Waller, Thomas Kapapa

**Affiliations:** 1 Institute for Anesthesiological Pathophysiology and Process Engineering, Medical Center, Ulm University, Helmholtzstraße 8/1, 89081 Ulm, Germany; tamara.merz@uni-ulm.de (T.M.); nicole.denoix@uni-ulm.de (N.D.); peter.radermacher@uni-ulm.de (P.R.); 2 Clinic for Psychosomatic Medicine and Psychotherapy, Medical Center, Ulm University, 89081 Ulm, Germany; 3 Department of Psychosomatic Medicine and Psychotherapy, Nuremberg General Hospital, Paracelsus Medical University, 90471 Nuremberg, Germany; christiane.waller@klinikum-nuernberg.de; 4 Clinic for Neurosurgery, Medical Center, Ulm University, 89081 Ulm, Germany; Thomas.Kapapa@uniklinik-ulm.de

**Keywords:** early life stress, adverse childhood experiences, posttraumatic stress disorder, traumatic brain injury, acute subdural hematoma, hemorrhagic shock, cystathionine-γ-lyase, hydrogen sulfide, oxytocin, pig

## Abstract

This paper explored the potential mediating role of hydrogen sulfide (H_2_S) and the oxytocin (OT) systems in hemorrhagic shock (HS) and/or traumatic brain injury (TBI). Morbidity and mortality after trauma mainly depend on the presence of HS and/or TBI. Rapid “repayment of the O_2_ debt” and prevention of brain tissue hypoxia are cornerstones of the management of both HS and TBI. Restoring tissue perfusion, however, generates an ischemia/reperfusion (I/R) injury due to the formation of reactive oxygen (ROS) and nitrogen (RNS) species. Moreover, pre-existing-medical-conditions (PEMC’s) can aggravate the occurrence and severity of complications after trauma. In addition to the “classic” chronic diseases (of cardiovascular or metabolic origin), there is growing awareness of psychological PEMC’s, e.g., early life stress (ELS) increases the predisposition to develop post-traumatic-stress-disorder (PTSD) and trauma patients with TBI show a significantly higher incidence of PTSD than patients without TBI. In fact, ELS is known to contribute to the developmental origins of cardiovascular disease. The neurotransmitter H_2_S is not only essential for the neuroendocrine stress response, but is also a promising therapeutic target in the prevention of chronic diseases induced by ELS. The neuroendocrine hormone OT has fundamental importance for brain development and social behavior, and, thus, is implicated in resilience or vulnerability to traumatic events. OT and H_2_S have been shown to interact in physical and psychological trauma and could, thus, be therapeutic targets to mitigate the acute post-traumatic effects of chronic PEMC’s. OT and H_2_S both share anti-inflammatory, anti-oxidant, and vasoactive properties; through the reperfusion injury salvage kinase (RISK) pathway, where their signaling mechanisms converge, they act via the regulation of nitric oxide (NO).

## 1. Introduction Polytrauma–Hemorrhage and Brain Injury

The presence of hemorrhage and traumatic brain injury (TBI) are the main determiners of morbidity and mortality after poly-trauma. Hemorrhage alone is responsible for 30–40% of the mortality [1,2,3] and also decisively determines the extent of post-traumatic multi-organ failure (MOF) [4,5]. In a prospective clinical study, it was shown that patients who required transfusions in the context of a trauma due to hemorrhage had a significantly increased risk of developing MOF [6]: 30% of the patients with systolic hypotension (<90 mmHg), metabolic acidosis (base excess −6 mmol/L), and/or requiring red blood cell transfusion within the first 12 h developed MOF within 2–3 days [6]. The underlying pathophysiological mechanism is systemic [2,3] and, in TBI, also local hyper-(neuro-)inflammation [7,8], which originates, in addition to direct mechanical/physical trauma, from tissue hypoxia due to blood loss and reduced perfusion [9]. TBI significantly worsens the acute prognosis of patients with polytrauma: for example, a retrospective long-term analysis over 15 years showed that TBI was responsible for 58%, whereas hemorrhagic shock (HS) was responsible for 28% of deaths after major trauma [10]. Moreover, long-term recovery of polytraumatized patients with TBI is much worse than in the absence of TBI; patients with TBI showed significantly worse functional restitution than patients with the same severity of trauma without TBI [11,12]. These functional impairments were associated with chronic systemic hyper-inflammation and increased signs of oxidative stress over the long-term course [13]. As mentioned above, shock-induced tissue hypoxia is a main trigger of hyper-inflammation, ultimately leading to MOF [9,14]. Therefore, rapid “repayment of the O_2_ debt” [15,16] and prevention of brain tissue hypoxia [17,18] to restore/maintain tissue O_2_-supply and thereby ATP-homeostasis are cornerstones of the management of TBI and HS. However, restoring tissue perfusion represents an ischemia/reperfusion (I/R) injury due to the formation of reactive oxygen (ROS) and nitrogen (RNS) species [19], which may further aggravate MOF as a result of ROS- and RNS-induced mitochondrial dysfunction [20,21,22]. This effect, as well as enhanced inflammation, may be further enhanced by catecholamines, which represent standard practice to maintain perfusion pressure [23,24,25].

## 2. Impact of Chronic Cardiovascular and Psychological Pre-Existing Medical Conditions on the Long-Term Patient Outcome

The presence and severity of pre-existing-medical-conditions (PEMC’s) critically influences morbidity and mortality [26,27], e.g., patients with cardiovascular disease (atherosclerosis, arterial hypertension or coronary artery disease) are at increased risk of post-traumatic MOF by a factor of 2–10 [28,29,30]. Vascular comorbid patients are characterized by chronic hyper-inflammation, excess ROS formation, and mitochondrial dysfunction [31,32] and, accordingly, patients with underlying cardiovascular co-morbidity (hypertension, coronary artery disease (CAD), congestive heart failure) present with a several-fold higher risk of MOF and mortality after HS and/or TBI [28,30]. This is in line with the worse outcome of TBI in the elderly [33], which in mice was shown to result from more pronounced oxidative stress [34].

Psychological trauma or early life stress (ELS) have been shown to have similar effects to these somatic pre-existing conditions [35]. ELS or adverse childhood experience (ACE) (i.e., trauma, neglect, etc. in childhood and/or adolescence) are of particular importance [35,36]. ELS/ACE increase the predisposition to develop post-traumatic stress disorder (PTSD) [36], and trauma patients with TBI show a significantly higher incidence of PTSD than patients without a TBI [37,38]. In this regard, the effect of ELS/ACE on long-term morbidity after TBI is similar to that of comparable experiences in adulthood [35]. These clinical-epidemiological data are supported by experimental data in rats: ELS/ACE induce chronic neuro-inflammation [39,40] and oxidative stress concomitant with reduced mitochondrial activity [41]. Acute TBI in addition to pre-traumatic ELS/ACE amplifies microglial activation, neuro-inflammation [42,43], and cortical atrophy [44]. The few available clinical data showed a direct relationship between PTSD severity and changes in cerebral cortex thickness in war veterans with/without ELS/ACE experience [45]. Other authors found a significant association between PTSD severity and late neuro-psychological damage after mild TBI, but no relationship with “white matter” integrity [46].

In addition to these direct effects of an ELS experience on the course after TBI, an indirect influence on morbidity and mortality after TBI can also be assumed in the context of the aforementioned importance of PEMC’s: it has long been known that ELS experiences increase the incidence and severity of chronic diseases [47,48,49,50,51,52,53], such as cardiovascular disease [54,55,56,57], chronic obstructive pulmonary disease (COPD) [58,59], or metabolic syndrome [60].

Psychological stress, in general [61,62,63], like physical trauma, and ELS/ACE, in particular, lead to a pro-inflammatory immune response in peripheral blood mononuclear cells (PBMC) [64] and granulocytes [65]; in addition, ELS/ACE amplify this pro-inflammatory response after acute stress exposure [66]. Psychological stress [67,68,69] and ELS/ACE are furthermore associated with increased oxidative and nitrosative stress [70,71,72]. Oxidative and nitrosative stress, in turn, leads to the uncoupling of electron transfer and transmembrane H^+^ transport and, thus, to the inhibition of the mitochondrial respiratory chain, which is considered as an essential mechanism for the development of MOF after trauma or in sepsis [22]: both animal data [20] and clinical studies [21] showed a direct correlation between morbidity and mortality on the one hand and the degree of inhibition of respiratory chain complex I on the other. Hyperinflammation further impairs the respiratory chain through the increased release of nitric oxide (NO) and its inhibitory effect on respiratory chain complex IV (i.e., cytochrome c oxidase) [73].

In turn, an altered mitochondrial function has also been attributed a specific role in the stress response in general [74,75] and specifically in ELS/ACE [76]: hyperinflammation and oxidative stress are associated with ELS/ACE and were accompanied by reduced mitochondrial respiratory chain activity of immune cells [70,77]. These associative clinical data regarding ELS/ACE, radical stress, and mitochondrial function are supported by mechanistic experimental findings: ELS led to the increased release of superoxide radical in mouse models with consecutively impaired endothelial cell function [78]. Moreover, in mice, the neuro-endocrine, metabolic, and inflammatory response to acute mental stress was shown to be determined by mitochondrial respiratory chain function [79]. Finally, a reciprocal relationship between TBI and psychological stress was demonstrated in rats: post-traumatic behavioral disturbances were directly related to mitochondrial dysfunction [80], and repetitive psychological stress in turn amplified the effect of TBI on mitochondrial respiratory chain protein expression [81].

## 3. The Role of Oxytocin in Psychological and Physical Trauma

**Figure 1:**
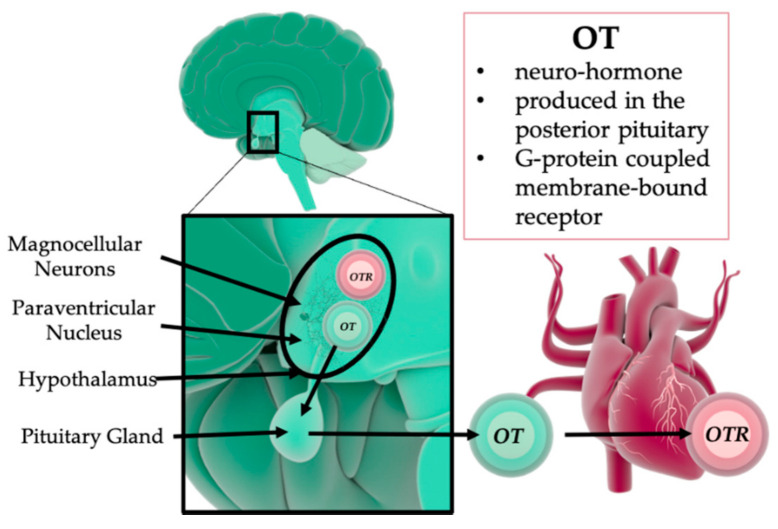
Oxytocin (OT) production and release. The neuro-hormone OT is produced within the magnocellular neurons of the hypothalamus and paraventricular nucleus (PVN). From the posterior lobe of the pituitary gland, OT is released into the circulation, where it acts via the G-protein-coupled oxytocin receptor (OTR). OT: oxytocin; OTR: oxytocin receptor; PVN: paraventricular nucleus. Illustrations of the heart, brain, and the circle shapes (spheres) were taken from the Library of Science and Medical Illustrations (somersault18:24, https://creativecommons.org/licenses/by-nc-sa/4.0/).

The neuro-hormone oxytocin (OT) plays a central role in the response to ELS/ACE. OT is produced in the hypothalamus and released from the posterior pituitary lobe (see Figure 1).

In addition to its well-known function in the transition to motherhood (inducing uterine contraction during labor, birth process, and the onset of lactation), OT is of fundamental importance for the development of the fetal brain and subsequent social behavior [82], which in response to traumatic events may manifest as resilience or vulnerability [83,84,85]. Indeed, patients with traumatic childhood experiences (CM) showed decreased expression of the OT receptor (OTR) in PBMC, which is necessary for OT-mediated responses [86,87]. Experimental findings show an alteration of cerebral OT concentrations and OTR expression in mice after ELS/ACE. These findings are complemented by a recent meta-analysis showing a decreased response to intranasal OT in human subjects with severe ELS/ACE history [88]. The OT system has also been implicated in the regulation of the immune system [89,90], both directly and indirectly via the balance between sympathetic and parasympathetic activity in the autonomic nervous system and through the “gut-brain axis” [91], as well as having antioxidant properties [89,92]. It has pleiotropic effects and is expressed in numerous organ systems: the gastrointestinal tract [93], kidney [94], heart [94,95], and the cardiovascular system, wherein it has been shown to be cardioprotective [96,97,98,99] by improving glucose utilization [100,101], stimulating the NO system [102,103], and having negative chronotropic effects [103]. Finally, in mice, OT-induced attenuation of depressive behavior, which was induced by ELS/ACE (maternal separation), was accompanied by improvement in hippocampal mitochondrial respiration [104].

However, the role of OT in circulatory shock has not been elucidated to date: OT activates not only OTR but also arginine vasopressin (AVP) receptors, as the two hormones only differ by two amino acids. Moreover, the activation of the respective receptors by OT and AVP is reciprocal [105,106]. Given the fact that endogenous AVP release plays a critical role in the regulation of blood pressure and volume in circulatory shock [107,108] and that AVP and its analogues are also used exogenously for hemodynamic management of shock [109,110,111,112,113], a corresponding role for OT can be assumed. However, except for the use of OT for uterine contraction in so-called atonic uterine hemorrhage or postpartum hemorrhage [114], few corresponding clinical and/or experimental studies are available [115,116,117]. Nevertheless, due to the above-mentioned multiple pleiotropic effects, OT is also referred to as “Nature’s Medicine” [118]. A clinical trial of intranasal OT has already been conducted in a pilot study in PTSD patients (Clinical Trials Registry NCT03238924: “Prolonged Exposure and Oxytocin”) [119]. Furthermore, OT is being reviewed in a consecutive multicenter study (Clinical Trials Registry NCT04228289: “Oxytocin to Treat PTSD”) [120]. Effects of intranasal OT application on hyperoxia-induced inflammation and oxidative stress induced by breathing hyperoxic gas mixtures during exercise are currently the subject of a US Navy study in healthy volunteers (Clinical Trials Registry NCT04732247: “Oxytocin for Oxidative Stress and Inflammation”). In this context, it should be noted that the actual presence of ELS/ACE may be of particular importance for the effectiveness of intranasal OT application: if pigs were repetitively treated immediately postnatal with intranasal OT, stress tolerance was actually worsened. OT-treated pigs showed more aggressive behavior in social interactions and a dysregulated HPA axis responsiveness at later time points, contrary to the original hypothesis of OT-induced long-term protective effects against social stress [121].

## 4. The Role of Hydrogen Sulfide in Psychological and Physical Trauma

It has been known for more than two decades that the three so-called “gaseous mediators” NO, carbon monoxide (CO), and hydrogen sulfide (H_2_S) play an essential role in the neuroendocrine stress response [122]. Gaseous mediators are endogenously synthesized by different enzyme systems [123,124]. Due to their physicochemical properties as gases and their very low molecular weight, and hence their freely diffusible properties, they have ubiquitous biological effects without the need for membrane-bound receptors and/or transport systems [124].

**Figure 2:**
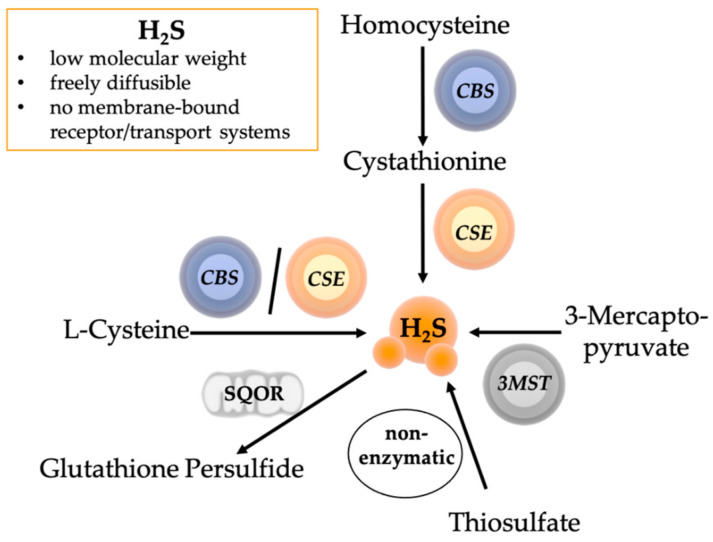
Hydrogen Sulfide (H_2_S) production and oxidation. H_2_S has a low molecular weight and is thus freely diffusible and acts independent of a membrane-bound receptor/transport system. H_2_S is produced enzymatically by three different enzymes: cystathionine γ-lyase (CSE), cystathionine-β-synthase (CBS), and 3-mercaptopyruvate sulphurtransferase (3MST). L-Cysteine is converted by CBS or CSE to H_2_S. Homocysteine is converted by CBS to cystathionine, which is then converted by CSE to H_2_S. Thiosulfate is an oxidation product of H_2_S, which is part of the stepwise enzymatic oxidation pathway within the mitochondria and can be utilized for non-enzymatic H_2_S production. In the mitochondria, H_2_S is oxidized by the sulfide:quinone oxidoreductase (SQOR) to glutathione persulfide. H_2_S: hydrogen sulfide; CSE: cystathionine γ-lyase; CBS: cystathionine-β-synthase; 3MST: 3-mercaptopyruvate sulphurtransferase; SQOR: sulfide:quinone oxidoreductase. Illustrations of the mitochondrion, the circle shapes, and spheres were taken from the Library of Science and Medical Illustrations (somersault18:24, https://creativecommons.org/licenses/by-nc-sa/4.0/).

H_2_S, which was first described as a “gaseous mediator” in the brain, plays a special role in the context of the neuroendocrine stress response [125]. Endogenously, H_2_S is synthesized by the enzymes cystathionine-γ-lyase (CSE), cystathionine-β-synthase (CBS), and 3-mercaptopyruvate sulfur transferase (3-MST) [123,124] (see Figure 2).

Genetic CSE deletion (CSE^−/−^) leads to the development of arterial hypertension [126]. In line with the CSE^−/−^-related development of arterial hypertension, we showed that CSE^−/−^ mice undergoing pre-traumatic cigarette smoke exposure to induce COPD presented with higher mean arterial pressures (MAP) during the acute phase after blunt chest trauma despite more pronounced metabolic depression as evidenced by reduced capacity to maintain normoglycemia [127]. We previously also showed that CSE expression is crucial for the adaptive response during acute stress situations [128,129]: (i) CSE-expression was inversely related to barrier dysfunction and, hence, the severity of sepsis-induced acute kidney injury (AKI) [130]; (ii) acute stress-related hyperglycemia down-regulated CSE expression, thereby impairing mitochondrial respiration [131]; (iii) CSE^−/−^ mice presented with aggravated post-traumatic acute lung injury (ALI) after pre-traumatic cigarette smoke exposure [127]; (iv) reduced CSE expression was associated with impaired mitochondrial respiration during sepsis-induced acute kidney injury [132]; (v) in resuscitated murine blunt chest trauma and HS, genetic mutation of another, mainly mitochondria-located H_2_S-producing enzyme 3-MST, the deletion of which is associated with hypertension and cardiac hypertrophy in aged mice [133], caused down-regulation of cardiac CSE expression, which coincided with lower activity of the mitochondrial complex IV activity [134]. Therapeutic effects of H_2_S are at least in part related to improved mitochondrial respiratory activity [135,136,137]. In fact, at low concentrations, H_2_S can indeed stimulate mitochondrial respiration by entering the respiratory electron transfer chain via the sulfide:quinone oxidoreductase (SQOR) (see Figure 2) and complex II, while at high concentrations it can inhibit mitochondrial respiration due to the inhibition of cytochrome c oxidase (complex IV) [138]. We previously showed using Na_2_S that the dose-effect relation of this biphasic H_2_S activity is cell-type dependent [139] and that H_2_S-related mitochondrial protection may depend on temperature and the presence/absence of circulatory shock [140,141,142]. In cultured cortical neurons from fetal rat brains, sodium thiosulfate, Na_2_S_2_O_3_, (STS), which can produce H_2_S both enzymatically and non-enzymatically [143,144] (see Figure 2), showed a similar U-shaped effect on mitochondrial respiration [145].

In addition, H_2_S was also shown to be beneficial in various models of TBI [14,146,147,148] by attenuating brain edema and maintenance of the blood-brain-barrier, which was at least in part related to improved mitochondrial function [136]. Despite various promising reports [19,149,150,151,152,153,154,155] exogenous H_2_S during HS produced equivocal results [156,157,158]. Undesired side effects were due to the narrow timing and dosing window [159] and the potentially high H_2_S peak concentrations [160] when the H_2_S-releasing salts NaSH and/or Na_2_S were used, or the aggravation of shock due to the vasodilatory properties of so-called slow-releasing H_2_S donors [161]. The latter problems may be overcome by evaluating already approved drugs, especially for potential clinical use, such as ammonium tetrathiomolybdate (approved for the treatment of Wilson’s disease) [155,162] or STS, a drug devoid of major undesired side effects and approved as an antidote for cyanide and mustard gas poisoning, cis-platinum overdose in oncology, and calciphyllaxy in end-stage kidney disease [163]. Ammonium tetrathiomolybdate prevented organ failure and morphological damage after cerebral and myocardial ischemia and hemorrhagic shock in mice [155,162]; however, none of the experimental groups received standard clinical therapy. Experimental data are also available for STS in organ protection after burns [164], myocardial infarction [165], and *E. coli* septicemia [166]. More recently, STS has been shown to be beneficial in LPS- and polymicrobial sepsis-induced ALI [167], acute liver failure [168], I/R injury [145], and Pseudomonas aeruginosa-sepsis [169] as well as both LPS- [170] and I/R-induced [171] brain injury. STS also protected against arterial hypertension-induced congestive heart failure [172,173] and kidney disease [174,175]. In good agreement with the findings on acute therapeutic efficacy of STS, we demonstrated that it attenuated ALI after HS in swine with coronary artery disease and, hence, consecutively reduced CSE expression [150]. This organ-protective property of STS under conditions of reduced CSE expression was confirmed by the even more pronounced effect in CSE^−/−^ mice after combined blunt chest trauma + HS [176].

## 5. Interaction of Oxytocin and Hydrogen Sulfide in Physical and Psychological Trauma

**Figure 3:**
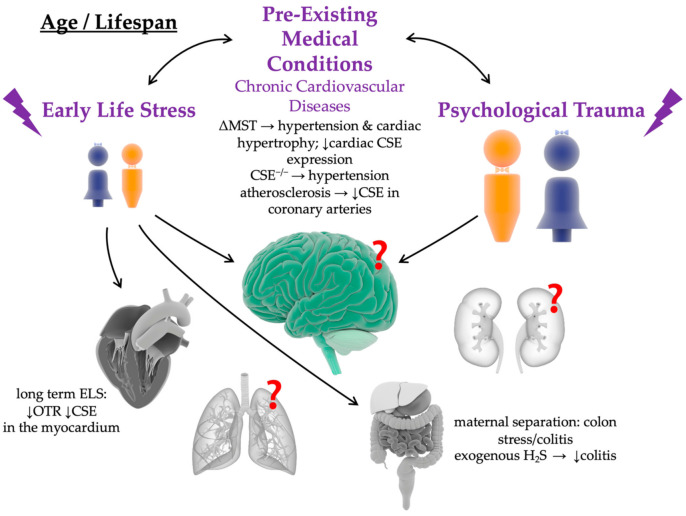
Interaction of Oxytocin/Oxytocin Receptor (OT/OTR) and Hydrogen Sulfide (H_2_S) in Early Life Stress (ELS) and Psychological Trauma. ELS can lead to the development of pre-existing medical conditions, such as chronic cardiovascular diseases. Both ELS and pre-existing medical conditions are associated with a dysregulation of the OT and H_2_S system in the heart and the colon. How the two systems are affected in other peripheral organs, or the brain is unknown so far. H_2_S: hydrogen sulfide; CSE: cystathionine γ-lyase; CBS: cystathionine-β-synthase; 3MST: 3-mercaptopyruvate sulphurtransferase; ΔMST: genetic mutation of 3MST; OTR: oxytocin receptor. ↓ slightly down. Illustrations of the male, female, brain, heart, lung, kidneys, gut, and liver were taken from the Library of Science and Medical Illustrations (somersault18:24, https://creativecommons.org/licenses/by-nc-sa/4.0/).

**Figure 4:**
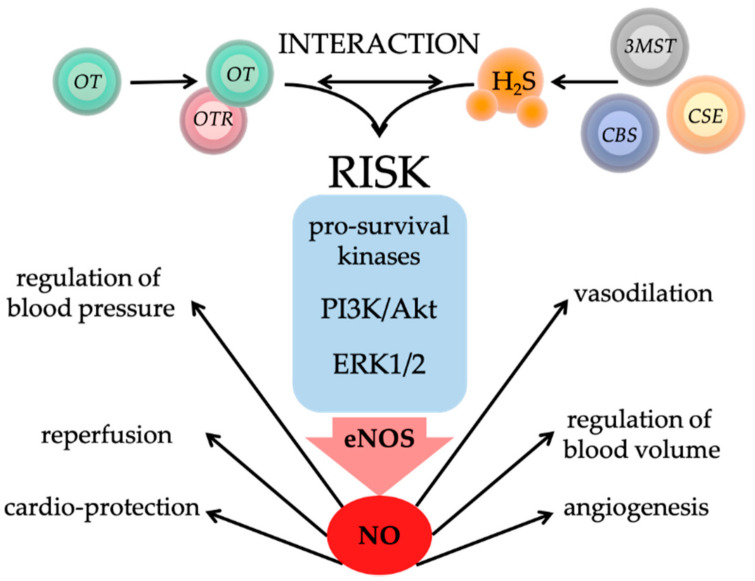
Interaction of Oxytocin/Oxytocin Receptor (OT/OTR) and Hydrogen Sulfide (H_2_S) via the Reperfusion Injury Salvage Kinase (RISK) Pathway. Through OT binding to the OTR, and/or H_2_S production from cystathionine γ-lyase (CSE), cystathionine-β-synthase (CBS), or 3-mercaptopyruvate sulphurtransferase (3MST), pro-survival kinases of the RISK pathway can be activated: Phosphatidylinositol 3-kinase/ Protein Kinase B (PI3K/Akt) and extracellular signal-regulated kinase 1/2 (ERK1/2). These kinases stimulate endothelial nitric oxide synthase (eNOS) and subsequently the release of nitric oxide (NO). NO activates regulation of blood pressure and blood volume, reperfusion, vasodilation, angiogenesis, and finally cardio-protection. H_2_S: hydrogen sulfide; CSE: cystathionine γ-lyase; CBS: cystathionine-β-synthase; 3MST: 3-mercaptopyruvate sulphurtransferase; OT: oxytocin; OTR: oxytocin receptor; RISK: reperfusion injury salvage kinase; PI3K: Phosphatidylinositol 3-kinase; Akt: Protein Kinase B; ERK1/2: extracellular signal-regulated kinase 1/2; eNOS: endothelial nitric oxide synthase; NO: nitric oxide. Illustrations of the circle shapes and spheres were taken from the Library of Science and Medical Illustrations (somersault18:24, https://creativecommons.org/licenses/by-nc-sa/4.0/).

**Figure 5:**
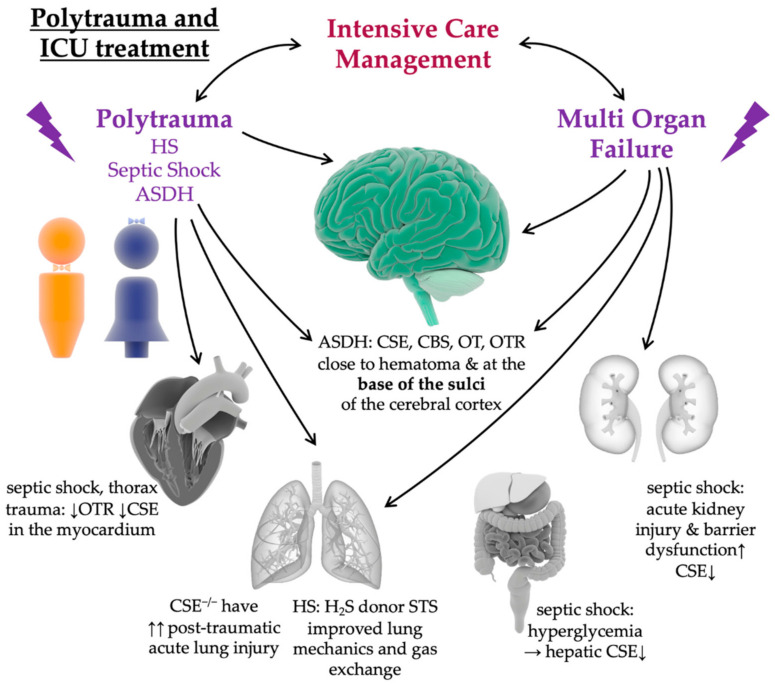
Interaction of Oxytocin/Oxytocin Receptor (OT/OTR) and Hydrogen Sulfide (H_2_S) in Polytrauma. Polytrauma, including hemorrhagic shock (HS), septic shock, and brain injury, such as acute subdural hematoma (ASDH), are associated with a dysregulation of the OT and H_2_S systems in the in the brain and the peripheral organs. Polytrauma can lead to multi-organ failure. Intensive care management is standard in the clinical treatment of polytrauma patients and provides organ-protective support. HS: hemorrhagic shock; ASDH: acute subdural hematoma; STS: sodium thiosulfate; H_2_S: hydrogen sulfide; CSE: cystathionine γ-lyase; CBS: cystathionine-β-synthase; CSE^−/−^: CSE knock out mice; OT: oxytocin; OTR: oxytocin receptor. ↓ slightly down, ↑ slightly up, ↑↑ strongly up. Illustrations of the male, female, brain, heart, lung, kidneys, gut, and liver were taken from the Library of Science and Medical Illustrations (somersault18:24, https://creativecommons.org/licenses/by-nc-sa/4.0/).

Recent findings show that H_2_S and the OT systems also interact in psychological trauma: Exogenous H_2_S delivery increased systemic AVP and OT concentrations [177]. Vitamin B deficiency-induced hyperhomocysteinemia with consecutively reduced endogenous H_2_S availability enhanced chemically induced experimental colitis [178], and ELS/ACE-induced colitis was significantly ameliorated by exogenous H_2_S supplementation [179] (see Figure 3). Moreover, OT [92,96] and H_2_S [180,181] showed comparable protective properties in the cardiovascular system and converge in the reperfusion injury salvage kinase (RISK) pathway, a signaling mechanism that acts via the regulation of NO [54,96,134] (see Figure 4). Our own findings support this interaction between OT (or the OTR) and H_2_S (and the CSE responsible for endothelial H_2_S formation [182]) Both blunt thoracic trauma [183] and hemorrhagic shock [134] were associated with a parallel reduction of OTR and CSE expression in the myocardium in mice (see Figure 5). In mice with a genetic CSE deletion, this reduction of OTR expression was markedly enhanced; in contrast, administration of the slow-releasing H_2_S donor GYY4137 was able to partially restore this effect [183]. A reciprocal interaction between CSE and OTR was demonstrated in mice with genetic deletion of OTR and, furthermore, ELS/ACE induced by maternal separation led to the reduction of myocardial CSE expression in comparison with wild type, and the authors showed that there was a linear relationship between myocardial OTR and CSE expression [85] (see Figure 3). The reciprocal interaction between OTR and CSE could also be confirmed in large animal experiments: pigs with coronary heart disease (CHD) showed a parallel reduction of myocardial expression of OTR and CSE after septic shock [184,185] (see Figure 5).

In the recently established large-animal model of acute subdural hematoma (ASDH) [186], co-localization of CSE, CBS, OT, and OTR, especially in the area of the hematoma and at the base of the sulci of the cerebral cortex [187], which is particularly vulnerable to intra cranial pressure (ICP) elevations (e.g., in the context of TBI), was demonstrated [188] (see Figure 5). This observation again highlights the importance of the interaction of the H_2_S and OT systems in the context of acute changes in blood volume, circulatory shock, and acute brain injury [189] (see Table 1).

## 6. Therapeutic Potential of Oxytocin and Hydrogen Sulfide in Trauma

The impaired endogenous availability of OT or H_2_S can theoretically be corrected by exogenous supply. As already mentioned above, for the exogenous supply of H_2_S, salts that directly release the molecule (NaSH, Na_2_S), slow releasing H_2_S donors (e.g., GYY4137, AP39), and already approved drugs (e.g., ammonium tetrathiomolybdate, STS) are available. H_2_S-releasing salts, injected as bolus intravenously (i.v.), can lead to toxic peak concentrations, which subsequently subside very rapidly and are barely detectable [128]. Moreover, these high peak concentrations have pro-inflammatory and oxidative effects [160], which are dose-dependent with possibly irreversible inhibition of mitochondrial respiration [138,190]. Even when these peak concentrations are avoided by continuous i.v. infusion, these H_2_S-releasing salts have a very small dose and time window, making them unsuitable for clinical use [141,142,159]. The slow-releasing H_2_S donors GYY4137 or AP39, which have been investigated so far in pre-clinical models in vivo, will in all likelihood not be considered for clinical use, as they either showed no organ-protective effects at all or possibly even pronounced undesirable side effects, despite the anti-inflammatory and oxidative effects mentioned above [161,191].

For exogenous delivery of OT, intranasal (e.g., for PTSD; [119] or multicenter study Clinical Trials Registry NCT04228289: “Oxytocin to Treat PTSD”) [120]), and i.v. administration can be considered, which in turn is used in obstetrics for contraction of the uterus and thus for prophylaxis of postpartum hemorrhage [192], especially after caesarean section [193]. Both forms of administration allow cerebral accumulation of OT [194,195], with identical doses in monkeys resulting in comparable or even higher concentrations in cerebrospinal fluid (CSF) after i.v. administration [194,196].

Nevertheless, the OT system is associated with stress-related responses, anxiolytic effects, maternal behavior, optimistic-belief updating, optimism and social reward perception, and several psychiatric disorders as well as prosocial (or anti-social) behaviors. [197,198,199,200]. Even though the role OT administration has been extensively studied, there is still ambivalence and a lack of clarity to the impact of OT treatment. OT administration was associated with pro-social behavior when the environment was considered safe and with defensive, anti-social behavior when the environment was perceived as unsafe [201]. The authors also suggest that OT treatment in individuals with a history of child maltreatment, borderline personality disorder, and/or severe attachment disorder, to promote aggressive tendencies [201]. Ellis et al., in a recent meta-analysis, concluded that individuals growing up in adverse conditions have lower endogenous OT levels and higher levels of methylation of the OTR gene [88]. Interestingly, individuals who reported less exposure to adverse childhood conditions responded more positively to intranasal OT administration [88]. The results of exogenous OT administration were ambivalent; on the one hand OT administration was anxiolytic in case with less severe forms of emotional trauma but on the other hand in patients with recent traumatic experience exogenous OT increased anxiogenic effects, enhancing the fear response [202,203]. The authors conclude that the use of exogenous OT for the prevention of PTSD warrants caution because of the ambivalent effects that appear to be context related [202]. The ambivalent findings and variable effects of OT administration in early life in individuals with ELS, PTSD, and/or psychiatric disorders suggests a need to better understand discrepancy between circulating levels of OT and OTR tissue expression levels (see Table 2).

## 7. Sex

The OT and H_2_S systems play sex-specific roles, and production of OT has been shown to vary between males and females [109]. The effect of ELS/ACE on the incidence or severity of subsequent COPD [204], as well as arterial hypertension, CHD, and cerebrovascular disease [205,206,207] was more pronounced in women, in contrast to the higher incidence of these conditions in men in the general population [208,209]. These epidemiological findings are complemented by recent data that the long-term outcome after TBI is worse in women than in men, particularly after “mild” TBI [210]. This indication of greater vulnerability of women to ELS/ACE is confirmed by experimental data in a model of early life adversity in pigs: female animals showed significantly more pronounced stress-induced pathophysiological changes in the gastrointestinal mucosa than males [211,212]. We previously showed that the aggravated posttraumatic pulmonary and systemic inflammation in CSE^−/−^ mice was more pronounced in male than in female animals [127]. This is in line with the most recent data that white matter damage and cognitive dysfunction was more pronounced in male than in female mice [213]. Moreover, mortality after TBI is most pronounced in the elderly, male patient [214,215], and, finally, the incidence and morbidity of ASDH is highest in this population [216,217]. However, there is clear evidence for age- and sex-dependent differences after murine TBI as well as the response to treatment: juvenile male mice revealed less acute inflammatory cytokine expression, but greater subacute microglial/macrophage accumulation, and improved neurological recovery after TBI [218]. This observation agrees with recent clinical findings that female patients showed worse long-term outcomes after mild TBI [210,219]. Finally, treatment with tranexamic acid to attenuate intracerebral hemorrhage after TBI attenuated blood-brain barrier disruption in males, but even increased its permeability in female mice [220], thus suggesting the importance of including sex in experimental protocols.

## 8. Impact of Intensive Care Treatment in Pre-Clinical Animal Models

Current guidelines of care for patients with TBI [17] include (i) control of ICP and related maintenance of cerebral perfusion pressure (CPP); (ii) avoidance of hypoxic phases, as assessed by the measurement of the partial pressure of O_2_ in cerebral tissue (Pt_c_O_2_), in addition to decompression by (hemi)craniectomy. Indeed, several clinical studies showed that ICP, CPP, and Pt_c_O_2_ are key determinants of both morbidity and mortality after TBI [221,222,223,224,225]. Additional prognostic factors after TBI include cerebral tissue concentrations of glutamate, glucose, lactate, and pyruvate [225,226,227,228], which indicate the metabolic state of the traumatized tissue.

However, none of the aforementioned studies integrated standard intensive care measures into the experimental protocol, limiting translational value. In fact, it may explain why in spite of many promising pre-clinical results the rodent acute brain injury models have been problematic in translation into clinical benefits [187,188].

In a randomized, controlled, double-blind trial conducted by our own group in pigs with CHD that underwent HS followed by 72 h of therapy according to the guidelines of the intensive care societies, a 24 h STS infusion (starting at the onset of intensive care in a “post-treatment” design) was associated with significant improvement in lung mechanics and gas exchange [150] (see Figure 5). Histomorphological and immunohistochemical analysis of tissue samples taken post-mortem from the paraventricular nuclei (PVN) of the hypothalamus showed that (i) HS alone (i.e., without additional local brain damage) resulted in only minor, neuro-histopathological changes and only in the “white matter”; and (ii) i.v. administration of STS in situations without local brain damage had no effect on the expression of CSE, CBS, OT, OTR, and the GR [150,229]. This finding is most likely due to the blood-brain barrier (BBB) remaining intact despite the HS, which prevented the passage of STS into the brain [230]. This situation of HS alone, i.e., without additional local damage to the brain, is diametrically opposed to the situation of an ASDH: histomorphological and immunohistochemical examination of the brain after ASDH alone, i.e., even without systemic circulatory depression and, thus, reduced perfusion of the brain, were accompanied by disruption of the BBB in the area of ASDH [188]. Other authors were also able to demonstrate an antioxidant effect for STS in terms of protection against doxyrubicin-induced oxidative DNA strand breaks [231]. The conclusion that STS apparently exhibits organ-protective effects after traumatic hemorrhagic shock in the presence of reduced endogenous H_2_S availability was confirmed by corresponding findings in mice with genetic CSE deletion [176]. Therefore, STS (i) which is approved as an antidote for cyanide and mustard gas poisoning, cis-platinum overdose in oncology, and calciphyllaxia [163]; (ii) for which dose information is available both as bolus and continuous i.v. infusion in humans and for large animals are known and identical [150,232,233,234]; and (iii) which is almost free of side effects even in high doses [163,235] and is therefore being tested in a clinical trial in patients with myocardial infarction (Clinical Trials Registry NCT02899364: “Sodium Thiosulfate to Preserve Cardiac Function in STEMI (GIPS-IV)”) may prove to be a safe and efficacious therapy for ASDH and HS [235].

Finally, an interesting aside, though not directly related to the topic at hand but very relevant to the current global pandemic of Coronavirus disease 2019 (COVID-19) caused by SARS-CoV-2, are the recent reports of reduced H_2_S levels as “a hallmark of COVID-19” [169,236,237] and the therapeutical potential for H_2_S donors; especially STS, in this context, is beyond the scope of this perspective but has been recently reviewed [150,169,238,239,240].

## 9. Conclusions

Taken together, this perspective explored the role of the biological connection of the H_2_S and OT systems in polytrauma e.g., HS and TBI with a special emphasis on translational modeling. Translational models need to reflect the pathophysiology of the patient population as well as the standard intensive care therapy, which polytrauma patients receive. As emphasized, many of the promising pre-clinical results in rodent TBI models have failed to translate into clinical benefits, and an obvious omission is the lack of intensive care unit (ICU) measures in these models. This perspective also highlighted the significance of the H_2_S and OT systems and their dysregulation in PEMC’s, both physical and psychological, that may affect therapeutic management of polytrauma patients. Sex-related differences were shown to also contribute to the complexity of therapeutic efforts and are often lacking in the experimental design. In an effort to improve translational studies, clinically relevant large animal models reflecting the pathophysiology (comorbidities) of the patient population (male and female) handled with the appropriate intensive care measures are necessary. Thus, in that there are no clinical data available in trauma, for HS and acute brain injury for the already approved STS (devoid of undesired side effects), it may be a relevant candidate to test in large animal models for these potential clinical applications.

## Figures and Tables

**Table 1:** Summary of studies: Interaction of the H_2_S system and the OT/OTR system.

Author and Year	Species	Experimental Challenge/Trauma/ Treatment	Interaction of OT and H_2_S
Trautwein et al., 2021 [134]	Mice	Naïve ΔMST animals Hemorrhagic Shock wt Hemorrhagic Shock & Blunt Chest Trauma wt	Constitutive CSE & OTR in cardiomyocytes CSE & OTR↓ CSE & OTR↓ CSE &OTR↓↓
Wigger et al., 2020 [85]	Mice	Maternal Separation (Early Life Stress) LTSS (long) STSS (short)	CSE & OTR↓↓ CSE↓ & OTR↑↑
Flannigan et al., 2014 [178]	Rats (vs. wt)	Diet for 6 weeks: “B-Def” lacked vitamins B_6_, B_9_, and B_12_ Colitis induction: 1. Drinking water supplemented with dextran sodium sulfate 2. Intracolonic administration of the hapten dinitrobenzene sulfonic acid 3. IL-10–deficient mice intra-colonical diallyl disulfide administration	In 1., 2., and 3., diet-induced hyperhomocysteinemia ↑colitis diallyl disulfide administration: ↓severity of colitis IL-10-deficient mice on a normal diet had ↓colonic H_2_S synthesis, a 40% ↑serum homocysteine IL-10–deficient mice fed the vitamin B-deficient diet exhibited ↑↑colonic inflammation Administration of IL-10 to the IL-10–deficient mice restored colonic H_2_S synthesis ↓serum homocysteine
Li et al., 2017 [179]	Mice	Maternal Seperation (vs. control animals) intraperitoneal NaHS administration (vs. vehicle)	Maternal Separation led to: ↓Crypt lengths, ↓goblet cells per crypt, ↓glutathione peroxidase activity, ↑expression of thiobarbituric acid reactive substances, ↑inducible nitric oxide synthase mRNA, ↑IL-6, ↑TNFα ↑myeloperoxidase Administration of NaHS: counteracted negative effects of maternal separation
Mani et al., 2013 [180]	Mice CSE^−/−^ (vs. wt)	Knock out and atherogenic diet intraperitoneal NaHS administration (vs. PBS injection)	Early fatty streak lesions in the aortic root ↑Plasma levels of cholesterol, ↑low-density lipoprotein cholesterol Hyperhomocysteinemia ↑Lesional oxidative stress and adhesion molecule expression ↑aortic intimal proliferation CSE^−/−^ treated with NaHS: inhibited the accelerated atherosclerosis development
Merz et al., 2018 [183]	Mice CSE^−/−^ (vs. wt)	Native wt Blunt Chest Trauma (and cigarette smoke exposure (CS)) Blunt Chest Trauma CSE^−/−^ (& CS) Blunt Chest Trauma CSE^−/−^ and GYY4137 administration (and CS)	Constitutive OTR in cardiomyocytes OTR↓ OTR↓↓ OTR↑↑
Nußbaum et al., 2016 [184]	Swine (hypercholesteremic vs. sham animals)	Septic Shock	Systemic Troponin↑ ↓ Cardiac output Cardiac CSE↓
Merz et al., 2020 [185]	Swine (hypercholesteremic vs. sham animals)	Septic Shock	Cardiac OTR↓
Coletti et al., 2015 [177]	Rats	Water deprivation for 12 and 24 h intra cerebroventricular Na_2_S	24 h water deprivation: ↑Activity of sulfide-generating enzymes in the medial basal hypothalamus Na_2_S administration: ↓Water intake, ↑arginine vasopressin, OT and corticosterone in plasma, ↓medial basal hypothalamus nitrate/nitrite content
Denoix et al., 2020 [188]	Swine	ASDH	CSE, CBS, OTR, and OT were localized to: (i) Cortical neurons in the gyri and at the base of sulci, where pressure-induced injury leads to maximal stress in the gyrencephalic brain (ii) In the parenchyma at the base of the sulci (iii) microvasculature and pial arteries (iv) Resident and infiltrating immune cells.

For the purposes of this perspective review, a medline pubmed search of the following key words was performed: early life stress, adverse childhood experience, posttraumatic stress disorder, traumatic brain injury, acute subdural hematoma, poly-trauma, hemorrhagic shock, sodium thiosulfate (Na_2_S_2_O_3_), cystathionine-γ-lyase (CSE), cystathionine-β-synthase (CBS), Oxytocin, Oxytocin-receptor, arginin-vasopressin (AVP), arginin-vasopressin-receptor (AVP-R), oxidative stress, nitrosative stress, porcine. Abbreviations: H_2_S = hydrogen sulfide; OT = oxytocin; OTR = oxytocin receptor; CSE = cystathionine γ-lyase; CBS = cystathionine-β-synthase; 3MST = 3-mercaptopyruvate sulphurtransferase; ΔMST = genetic mutation of 3MST; CS = cigarette smoke exposure; NaHS = Sodium hydrosulfide; Na_2_S = Sodium sulfide; wt = wild type; CSE^−/−^ = CSE knock out; PBS = phosphate buffered saline; ASDH: acute subdural hematoma. ↓ slightly down, ↓↓ strongly down, ↑ slightly up, ↑↑ strongly up.

**Table 2:** Summary of studies: Therapeutic potential of the H_2_S system and the OT/OTR system in trauma.

Author and Year	Species	Experimental Challenge	Therapeutic Potential of OT and H_2_S in Trauma
Ellis et al., 2021 [88]	Humans	ELS Intranasally administered OT	People who grew up under more adverse conditions tend to have ↓endogenous OT Early adversity is associated with higher levels of methylation of the OTR gene Adults who report ↓levels of childhood adversity tend to show ↑positive responses to intranasal OT
Flanagan et al., 2018 [119]	Humans	Posttraumatic Stress Disorder (PTSD) Treatment: Prolonged Exposure Therapy and intranasal OT (vs. placebo)	OT group: ↓PTSD & depression symptoms during Prolonged Exposure Therapy ↑Working alliance scores
Bracht et al., 2012 [159]	Swine	Hemorrhagic Shock Intravenous Na_2_S administration 1. 2 h before hemorrhage 2. Simultaneously with blood removal 3. At the beginning of retransfusion of shed blood	2. simultaneous treatment group: ↓Progressive kidney, liver, and cardiocirculatory dysfunction ↓Histological damage of lung, liver, and kidney Na_2_S: ↓mortality irrespective of the timing of its administration
Whiteman et al., 2010 [160]	Murine RAW264.7 macrophages	Lipopolysaccharide (LPS) treatment NaHS or GYY4137 administration	GYY4137 led to: Concentration-dependently ↓LPS-induced release of proinflammatory mediators (IL-1β, IL-6, TNF⍺, NO, and PGE(2)), ↑synthesis of the antiinflammatory IL-10 NaHSlet to: Biphasic effect on proinflammatory mediators, at high concentrations, ↑synthesis of IL-1β, IL-6, NO, PGE(2) and TNF⍺
Wepler et al., 2019 [161]	Mice	Wave-induced thorax trauma and hemorrhagic shock (vs. sham) Intravenous bolus injection high and low dose of AP39 (vs. vehicle)	High-dose AP39 in thorax trauma: ↓Systemic inflammation, ↓inducible nitric oxide synthase and IκBα in lung tissue thorax trauma and hemorrhagic shock: High-dose AP39: ↓Mean arterial pressure, ↑norepinephrine requirements, ↑mortality Low-dose AP39: no effects
Matallo et al., 2014 [190]	Immortalized cell line (AMJ2-C11)	Na_2_S solution stimulation	Mitochondria analysis: The onset of inhibition of cell respiration by sulfide occurs earlier under a continuous exposure when approaching the anoxic condition.
Nußbaum et al., 2017 [191]	Swine (Pre-existing coronary artery disease)	Septic Shock (vs. sham) intravenous GYY4137 administration	GYY4137 led to: ↑Aerobic glucose oxidation, ↑requirements of exogenous glucose to maintain normoglycemia, ↓arterial pH, ↓base excess ↓Cardiac eNOS expression, ↑troponin levels no effect on cardiac and kidney function or the systemic inflammatory response
Lee et al., 2020 [194]	Rhesus Macaques	Labelled OT administration nebulizer/intravenous infusion/intranasal	2 h after OT administration: Labeled OT is found after intranasal administration in orbitofrontal cortex, striatum, brainstem, and thalamus (these lie in the trajectories of the olfactory and trigeminal nerves, bypassing the blood-brain barrier)
Martins et al., 2020 [195]	Humans	healthy volunteers OT administration nebulizer/intravenous infusion/standard nasal spray (vs. placebo or saline)	OT-induced: ↓Amygdala perfusion (a key hub of the OT central circuitry)due to OT ↑in systemic circulation following both intranasal and intravenous OT administration Robust evidence confirming the validity of the intranasal route to target specific brain regions
Lee et al., 2018 [196]	Rhesus Macaques	Labelled OT administration: intravenous infusion/intranasal (vs. intranasal saline as control)	Cerebro-spinal fluid penetrance of labelled OT exogenous OT delivered by intranasal and intravenous administration Intravenous administration of labelled OT did not lead to increased endogenous OT or endogenous OT in the cerebro-spinal fluid
Ma et al., 2016 [199]	Humans	Intranasally administered OT	↑Optimistic belief updating by facilitating updates of desirable feedback, but ↓updates of undesirable feedback ↑Learning rate (the strength of association between estimation error and subsequent update) of desirable feedback ↑Participants’ confidence in their estimates after receiving desirable but not undesirable feedback
Saphire-Bernstein et al., 2011 [200]	Humans	Genotype of OTR	Link between the OTR SNP rs53576 and psychological resources “A” allele carriers have ↓levels of optimism, mastery, and self-esteem, relative to G/G homozygotes
Domes et al., 2010 [203]	Humans	Presented with fearful, angry, happy and neutral facial expressions after a single dose of intranasal OT or placebo administration	Blood-oxygen-level-dependent signal was ↑in the left amygdala, the fusiform gyrus & the superior temporal gyrus in response to fearful faces & in the inferior frontal gyrus in response to angry and happy faces following OT treatment. independent of basal plasma levels of OT, estradiol, and progesterone

For the purposes of this perspective review a medline pubmed search of the following key words was performed: early life stress, adverse childhood experience, posttraumatic stress disorder, traumatic brain injury, acute subdural hematoma, poly-trauma, hemorrhagic shock, sodium thiosulfate (Na_2_S_2_O_3_), cystathionine-γ-lyase (CSE), cystathionine-β-synthase (CBS), Oxytocin, Oxytocin-receptor, arginin-vasopressin (AVP), arginin-vasopressin-receptor (AVP-R), oxidative stress, nitrosative stress, porcine. Abbreviations: ELS = early life stress; PTSD = post-traumatic stress disorder; H_2_S = hydrogen sulfide; OT = oxytocin; OTR = oxytocin receptor; NaHS = Sodium hydrosulfide; Na_2_S = Sodium sulfide; SNP = single nucleotide polymorphism. ↓ slightly down, ↓↓ strongly down, ↑ slightly up, ↑↑ strongly up.

## Data Availability

Not applicable.
